# The influence of conscientiousness and context on the emotional response during understanding

**DOI:** 10.3389/fpsyg.2025.1589709

**Published:** 2025-12-16

**Authors:** Jenny Jun, Milan Lazic, Earl Woodruff

**Affiliations:** 1 Wisdom and Identity Lab, Department of Applied Psychology and Human Development, Ontario Institute for Studies in Education, University of Toronto, Toronto, ON, Canada; 2 Emotions and Learning Optimization Lab, Department of Applied Psychology and Human Development, Ontario Institute for Studies in Education, University of Toronto, Toronto, ON, Canada

**Keywords:** emotion, understanding, personality, multilevel modelling, iMotions

## Abstract

**Introduction:**

In this exploratory study, we use a logistic multilevel model to examine the interplay between personality, emotions, and understanding.

**Methods:**

The impact of Conscientiousness, a Big Five Personality trait, on emotional responses during riddle-solving attempts was investigated in 101 participants, who each tackled 15 riddles remotely via Zoom. Video recordings were analyzed using iMotions with AFFDEX to identify the emotion type (positive, negative, and epistemic) experienced during the riddle-solving (understanding) process.

**Results:**

The study revealed significant effects of both positive and negative emotions on understanding scores. Upon examining cross-level interactions, the effect of trait Conscientiousness on the association between emotion type and understanding scores was not significant. Our random intercepts model appeared to provide the most satisfactory explanation for our exploratory findings. Further, the model demonstrated good discrimination ability, as evidenced by our area under the Receiver Operating Characteristic (ROC) curve.

**Discussion:**

Our results highlight the fundamental role of context, specifically the individual’s perceived value of a learning activity and the nature of their understanding—emergent or established—in shaping the emotions evoked during the process of understanding. For future endeavors, we recommend a concentrated focus on the complex interplay between personality, emotions, and understanding, especially when understanding is in its emergent phase and the task employed for measurement provokes emotional investment.

## 1 Introduction

Learning is a dynamic and multifaceted process that encompasses a variety of objectives, such as acquiring knowledge, honing critical thinking skills, fostering socialization, and promoting personal growth. Arguably, the most crucial objective of learning is understanding. When “understanding” is achieved, learning delves beyond the mere memorization of facts, terms, and procedures (Mayer, 2002) and actively engages the processes that facilitate the development of innovative ideas, practical skills, and insightful perspectives (de Regt, 2017; Grimm, 2012). As it evolves, understanding provides valuable insights, offering solutions to problems, practical applications of knowledge, and connections between seemingly unrelated pieces of information. The significance of understanding has been demonstrated in its positive impact on a range of academic outcomes. For instance, students who learn with a focus on the process of understanding exhibit improved performance in coursework (Phan, 2009), state examinations, assessments of intricate problem-solving abilities, and mastery of essential content in reading, mathematics, and science (Zeiser et al., 2014). Additionally, these students report heightened levels of self-efficacy and motivation to learn when compared to their peers who do not prioritize understanding (Zeiser et al., 2014).

Most importantly, understanding is both a concept and a mental state (Grimm, 2012; Olson, 2022; Van Camp, 2014). This means that to understand, an individual must satisfy both the objective criteria for understanding relative to their current context and experience the subjective feeling of understanding (Olson, 2022). For example, a student studying the differences between general relativity and quantum mechanics may experience the emotional sensation of understanding, which can foster confidence and allow them to progress in their studies. However, this emotional experience alone is insufficient to determine that the student has indeed understood, as it is also essential for the student to meet an objective standard of correctness, such as accurately explaining the differences between general relativity and quantum mechanics to someone or on a test. In this scenario, the student’s physics teacher would evaluate their explanation, and if found to be accurate, affirm that the student understands.

Nevertheless, the emotions experienced before understanding—such as joy, confusion, and engagement—are as vital as the ultimate subjective sensation of understanding (Olson, 2022). While research on the direct influence of emotions within the context of understanding remains limited, there is considerable knowledge about the role of emotions in learning and the associated factors that affect the process toward understanding. Emotions influence the process of understanding by impacting the cognitive processes involved in learning (Immordino-Yang and Damasio, 2007). Generally, positively valenced emotions like hope and enjoyment support understanding by promoting the use of elaboration strategies (Schweder, 2019), flexible thinking, intrinsic motivation (D’Mello et al., 2017; Pekrun et al., 2011), and deep learning (Pekrun et al., 2017). In contrast, negatively valenced emotions such as anxiety and anger can lead to boredom and disengagement (D’Mello and Graesser, 2012; Pekrun et al., 2010), and encourage task-irrelevant thinking, preoccupation with failure (Gable and Harmon-Jones, 2010), surface learning (Pekrun et al., 2017), as well as reduced cognitive flexibility and motivation (Pekrun and Stephens, 2010). According to Pekrun et al. (2016), epistemic emotions such as curiosity, surprise, confusion, and the satisfaction associated with understanding are intricately woven into cognitive processes and significantly shape behaviours pertinent to learning and decision-making. They also manifest as varying effects on understanding (D’Mello and Graesser, 2012). For instance, when learners encounter obstacles that hinder their learning goals, such as information that contradicts their existing knowledge, confusion can arise. By actively working to resolve confusion (e.g., via active questioning), learners can achieve deep learning. However, if the impasse remains unresolved, confusion can escalate into frustration and eventually boredom, thereby impeding understanding.

The emotions experienced during the process of understanding can differ among individuals, depending on their sensitivity to and appraisal of their context, as well as their prior experiences and goals (Lazarus, 1999; Scherer and Moors, 2019). According to the Control-Value Theory (Pekrun, 2006; Pekrun and Perry, 2014), emotions that surface in the classroom are derived from two continuous appraisals: one concerning the learner’s perceived control over outcomes, and the other pertaining to the value assigned to the task and its outcome. Both appraisals take into account past experiences of success and failure, as well as expectations of future outcomes. For example, if a student encounters anxiety while taking a math exam and subsequently fails, both the experience of anxiety and the outcome of failure would impact their appraisals of perceived control and the value attributed to future math exams. These appraisals, in turn, influence their emotions and anticipated results. And if a student consistently experiences specific emotions (e.g., anxiety) and outcomes (e.g., failing), stable beliefs can develop, fundamentally shaping their sensitivity to and appraisals of such situations (e.g., “I am terrible at math exams, and they cause anxiety”).

Western educational curricula are designed to introduce students to new intellectual domains, often structuring instruction around the progressive acquisition of new knowledge—a process supported by psychological theories of learning (Vygotsky and Cole, 1978; Piaget, 1952; Bransford et al., 2000). Inquiry-based and discovery-driven models similarly prioritize exposure to novel challenges, reinforcing the idea that academic environments are deliberately structured to elicit cognitive and emotional responses to new information (Hmelo-Silver et al., 2007; Bruner, 1961).

In uncharted learning territory, where stable reference points are lacking, personality shapes how students appraise, emotionally engage with, and integrate new concepts, influencing their adaptation to novel learning challenges. Personality is a trait variable that highlights stable individual differences in cognition, motivation, and emotionality (Pekrun, 2006; Montag and Panksepp, 2017). The Five Factor Model of Personality (Big Five) represents a prominent research model, with each trait associated with emotional tendencies (McCrae and Costa, 2008). Conscientiousness, one of the Big Five traits, is characterized by a tendency toward organization, responsibility, and goal-directed behaviour. It is associated with self-discipline, perseverance, and a preference for planned over spontaneous actions (Roberts et al., 2017). Among the Big Five, conscientiousness plays a particularly significant role in learning outcomes and academic performance, often matching or even exceeding intelligence in predicting academic success (Poropat, 2009).

Conscientious individuals engage in structured study behaviours, manage time effectively, and maintain consistent effort, all of which directly enhance academic achievement (Bidjerano and Dai, 2007; Noftle and Robins, 2007). Their capacity to sustain attention, regulate impulses, and approach tasks systematically fosters deeper engagement with learning material and facilitates retention (Rimfeld et al., 2016). Beyond these direct effects, conscientiousness indirectly supports academic performance by reducing stress, promoting emotional regulation, and cultivating a balanced lifestyle—factors that sustain motivation and resilience in the face of challenges (De Feyter et al., 2012; Richardson et al., 2012). Because students frequently encounter uncharted learning territory, conscientiousness shapes how they appraise unfamiliar academic situations, influencing their emotional responses and, ultimately, their ability to integrate and understand new material.

While conscientiousness is strongly linked to academic performance and generally associated with positive emotions (Livingstone and Srivastava, 2014; Reisenzein et al., 2020), research on the specific achievement emotions most strongly linked to it is lacking. Furthermore, how these emotions influence understanding remains unclear. Clarifying this relationship is essential to understanding how conscientiousness influences performance and understanding in academic settings. If certain achievement emotions mediate this relationship, fostering conscientious-like behaviours through targeted emotional strategies may provide an alternative way to enhance engagement and understanding in less conscientious individuals.

This exploratory study examines whether individual differences in personality shape how emotion type relates to real-time understanding. Using a logistic multilevel model, we addressed three questions: (1) Do understanding scores vary between individuals? (2) Do understanding scores differ by emotion type at the within-individual level? (3) Does the association between emotion type and understanding vary by trait Conscientiousness? The following hypotheses were formulated:

(1) Understanding scores vary between individuals, reflected by nonzero between-person variance. (2) Understanding scores differ by emotion type, with positive emotions expected to increase the likelihood of understanding, negative emotions expected to decrease it, and confusion showing mixed or uncertain effects given its epistemic nature. Because this was an exploratory analysis, no specific hypothesis was proposed for the cross-level interaction.

## 2 Method

### 2.1 Participants

In this study, 101 participants (total observations = 1,314) were enrolled with a mean age of 24 years (*SD* = 4.29), of which 77 participants (75.4%) were female. We used a combination of recruitment strategies, including online platforms (e.g., Facebook) and student-affiliated websites (e.g., University of Toronto Psychology Student’s Association), as well as word-of-mouth referrals. Although most participants were currently enrolled in undergraduate programs, eligibility for participation was limited to individuals who were either current undergraduate students or had completed some undergraduate education to ensure a straightforward recruitment process.

### 2.2 Measures

#### 2.2.1 Understanding

Understanding was evaluated by requiring participants to solve a set of 15 riddles—a quantity chosen based on our preliminary study, suggesting it optimally balanced engagement and fatigue, while facilitating the collection of ample data. Riddles served as an effective metric for understanding, with participants scoring a 0 for an incorrect answer or 1 for a correct one. This approach was taken due to several compelling qualities of riddles: (1) they are inherently engaging, (2) universally comprehensible due to their cross-cultural presence, (3) necessitate core aspects of understanding (such as logical, lateral, and flexible thinking), and (4) elicit a range of emotions, thereby facilitating the study of emotional responses during understanding. Moreover, the selection of riddles was underpinned by the premise that most participants would not have established beliefs about riddle-solving (Pekrun, 2006). The riddles, sourced from Toplak et al. (2013), Bar-Hillel et al. (2018), and various online platforms, varied in difficulty and type—some were mathematical, while the majority were wordplay-oriented. Among the selection were more challenging riddles, e.g., Riddle 11 (see Appendix A), known to exploit cognitive biases and thus prove difficult to answer correctly—an effect explored by Toplak et al. (2013).

Finally, as care was taken to ensure that each riddle had one distinct answer and explanation (see Appendix A for the list of riddles used in this study), participants were not considered to understand unless they provided both the correct answer and justification—not only outlining the logical progression leading to the answer, but also demonstrating an understanding of the riddle’s inherent subtleties, ambiguities, and nuances, informed by the riddle’s wording, structure, or theme. For example, take the riddle: ‘I start with M, end with X, and have a never-ending amount of letters. What am I?’ The answer is ‘mailbox’. The corresponding justification involves explaining that ‘mailbox’ begins with the letter ‘M’, ends with the letter ‘X’, and is designed to house an indefinite quantity of letters, referring to postal mail. Requiring accurate justification ensured that cases where participants merely guessed the right answer would not be considered as understanding (Olson, 2022).

#### 2.2.2 Emotions

Emotions are defined here according to appraisal-based theories (Scherer and Moors, 2019; Pekrun, 2006), encompassing affective and epistemic states that involve goal-directed evaluations of learning events. Emotions were assessed using the AFFDEX module in the facial expression analysis suite developed by iMotions^1^ (iMotions, 2019) and validated in studies on static images and videos (Kulke et al., 2020). Although iMotions provides the option to measure emotions using both the AFFDEX and FACET modules, only AFFDEX was utilized in this study. This was due to unresolved post-processing difficulties with FACET within the iMotions system. However, it is important to note that a previous study by Dupré et al. (2020) found no significant differences in accuracy between the two modules when measuring spontaneous facial expressions. Therefore, the decision to use AFFDEX over FACET in this study did not result in any foreseen or significant limitations.

AFFDEX uses the Facial Action Coding System (FACS; Ekman and Friesen, 1978) to automatically code facial expressions of emotions in real-time using a computer camera. Changes in key facial landmarks (e.g., eyebrows, lips, and eyes) are measured and combined to classify seven emotions: anger, sadness, disgust, contempt, fear, joy, and confusion, which map onto three emotion types used in this study: positive, negative, and epistemic. Joy was treated as a positive emotion reflecting high-valence affective responses linked to fluent task engagement, whereas confusion was analyzed as a distinct epistemic emotion associated with cognitive disequilibrium and problem solving (Pekrun et al., 2016; D’Mello and Graesser, 2012). All 7 emotions were included in this study as they represent the three different emotion types of interest. AFFDEX provides values for frequency or the number of instances per frame (second) each emotion measured exceeds a predetermined likelihood threshold. On a scale ranging from 0 to 100, a threshold of ‘25’ represents a mild facial response, ‘50’ a moderately strong response, and ‘75’ a strong response (see text footnote 1). The likelihood threshold was set at 50 to enhance reliability in measuring emotions while also capturing relatively subtle expressions.

Facial expressions and emotions were recorded from the moment participants read a given riddle until they started providing a final answer and justification. To avoid skewing facial expressions and emotion data, participants were instructed not to speak out loud while solving riddles. Instead, they were provided with a textbox where they could type out their thoughts.

To evaluate the influence of negative emotions on understanding, we consolidated the values for several emotions: anger, sadness, disgust, contempt, and fear. This decision was a methodological compromise necessitated by the limited number of observations per emotion type and the exploratory scope of the study. Confusion and joy were the only epistemic and positive emotions examined, respectively. Given the disparity in the number of emotions within each emotional category, we decided to standardize these variables to improve their comparability.

Although VIF originates from linear regression, it remains a useful approximate diagnostic for multicollinearity among fixed-effect predictors in multilevel logistic models. We therefore computed VIFs for the emotion-type variables by regressing each predictor on the others. The average VIF was 1.55, indicating minimal collinearity, as values near 1 reflect negligible shared variance.

#### 2.2.3 Personality

Conscientiousness was measured using the Big Five Inventory 2 (BFI-2; Soto and John, 2017). The BFI-2 is a self-report personality assessment tool consisting of 60 items scored on a 5-point Likert scale (1 = strongly disagree, 5 = strongly agree), with 12 items measuring each trait. As a valid and reliable updated version of the original Big Five Inventory, the BFI-2 domains highly correlate with the BFI domains (correlation for Conscientiousness was 0.91). The BFI-2 was chosen over the BFI for this study as it preserves the original measure’s conceptual focus, comprehensibility, and conciseness, while enhancing reliability and validity by minimizing the influence of acquiescent responding (i.e., the tendency of individuals to consistently agree or disagree with questionnaire items irrespective of their content).

We standardized trait Conscientiousness prior to categorizing it into tertiles (T): low (reference group, T1), medium (T2), and high (T3). Standardization does not affect tertile assignment but places the underlying continuous variable on a common scale.

### 2.3 Statistical analysis

In this study, we adopted a repeated measures design and collected 1,314 binary understanding scores from 101 participants, yielding an average of 13 scores per participant.

To examine the association between understanding scores and emotion type, we estimated a two-level logistic multilevel model (MLM) in Stata/BE 18.0 using ‘melogit’. A multilevel approach was required because observations (Level-1, L1) were nested within individuals (Level-2, L2), violating the independence assumption of single-level logistic regression. MLM accounts for within-person dependence by estimating a random intercept for each participant. We confirmed that including random intercepts improved model fit over a single-level logistic model using a likelihood ratio test. MLM also enabled us to test a theoretically motivated cross-level interaction between emotion type (L1) and a person-level personality variable (L2). Finally, MLM allowed all analyses to be conducted on the raw repeated-measures data, avoiding aggregation and the associated loss of within-person information.

Before testing our primary hypothesis, we estimated the intraclass correlation coefficient (ICC) to quantify the proportion of latent-scale variance attributable to between-individual differences. We then estimated a null random-intercept logistic model to test our first hypothesis: whether allowing intercepts to vary across individuals improved model fit compared with a single-level logistic model. Likelihood ratio (LR) tests were used to evaluate whether the added random-effect variance significantly improved the model. A significant LR test indicates that the more complex multilevel specification provides a better fit than the simpler model.

Our second hypothesis was tested using a random-intercepts model with group-mean centered L1 predictors (positive, negative, and confusion emotion type). Group-mean centering is used to isolate within-individual associations and ensure that L1 effects are interpreted at the within-individual rather than between-individual level. To evaluate model improvement relative to the null model, we computed McFadden’s R-squared, a pseudo-R-squared metric for logistic regression that quantifies the proportional increase in log-likelihood from the intercept-only model to the fitted model with predictors. McFadden’s R-squared provides a single summary measure of relative model fit and is well suited for logistic models with repeated-measures data.

To test our third hypothesis—whether the association between emotion type and understanding scores varied by trait Conscientiousness—we examined cross-level interactions. Because Conscientiousness was categorized into tertiles, it was modelled as a three-level variable, yielding two coefficients comparing the medium and high tertiles to the low-tertile reference group. These interactions linked L1 emotion type with L2 Conscientiousness.

To verify our model, we compared the goodness of fit of two models using Akaike Information Criterion (AIC) and Bayesian Information Criterion (BIC). Both AIC and BIC are derived from the model likelihood and balance model fit and complexity by penalizing additional parameters. Lower values indicate better fit. AIC prioritizes model fit and tolerates additional parameters, making it useful for predictive modeling. BIC imposes a stronger penalty for complexity, especially with larger samples, favouring more parsimonious models. Given the modest number of parameters relative to the sample size, model complexity was acceptable for our analysis, although AIC and BIC primarily guide model selection rather than diagnose overfitting.

To evaluate the model’s ability to distinguish between events and nonevents, we estimated the Area Under the Receiver Operating Characteristic curve (AUC). Because AUC is a rank-based measure, clustering affects standard errors but not the point estimate, so we report the standard AUC, corresponding to Somers’ D (D = 0.592).

The dataset showed mild class imbalance (37.6% in the minority class), which is not severe enough to warrant Precision–Recall AUC; under these conditions, ROC-AUC remains appropriate.

### 2.4 Procedure

Participants completed this study remotely via Zoom. Before beginning the study, they were asked to display their student ID card or provide other proof of undergraduate enrolment. To ensure high-quality video data, participants were required to be centered in front of their camera with good facial lighting, among other criteria (e.g., no facial obstructions, a stable surface for their computer, no phones or tablets, no green-screen filters or movement in the background). Participants were then given access to a Qualtrics link where they first completed a consent form, demographic questionnaire, and BFI-2 before attempting to solve 15 riddles. As participants completed the consent form, questionnaires, and riddles, they shared their screens on Zoom, to monitor their progress and provide feedback throughout the study. To ensure consistency and neutrality of the emotional climate, the same researcher conducted each session, adhering to the same procedural script and feedback on the riddles. The ‘pin’ feature in Zoom was used to ensure consistent recording of participant faces. Video recording began when participants started working on the first riddle. The researcher’s video and audio were turned off to minimize influence on participants as they solved the riddles.

Participants were given a maximum of 3 min to answer each riddle, allowing sufficient time for reading, considering, and answering the riddle while being mindful of response fatigue. After 3 min, participants were asked to provide an answer and justification. Once an answer and justification were provided, verbal feedback was given, indicating whether the answer was correct or incorrect. Participants who answered incorrectly were given an opportunity to try again if they wished. When a participant was unable to provide a correct answer or declined to try again, the correct answer and justification were provided. This ensured that participants did not dwell on unsolved riddles while working on subsequent ones. Additionally, before moving on to the next riddle, participants were asked if they already knew the correct answer to the current riddle to determine whether to exclude response. After completing the riddles, participants were debriefed and compensated $10 via e-transfer. The Zoom recordings were then processed and analyzed in iMotions.

## 3 Results

### 3.1 Sample characteristics

The characteristics of our study population were calculated to illustrate the distributions in relation to the Big Five traits and emotion types, as presented in Table 1.

**Table 1:** Descriptive statistics: understanding scores, personality scores, and emotion type frequencies.

	Mean	SD	Median			
Understanding scores (%)	63.49	22.89	66.66			
BFI-2 trait conscientiousness scores	3.64	0.69	3.7			

Emotion type (frames per second)	Quartile 1	Quartile 2	Quartile 3	Quartile 4	Median	Mean
Positive	1,128	3,627	10,058	41,790	0	43.07
Negative	1,217	4,120	11,591	2,689	0	52.24
Confusion	223	862	2,689	11,937	0	11.95

Summary of understanding scores—average across individuals (%), BFI-2 trait conscientiousness scores, and emotion type frequencies (frames per second)—with means, standard deviation, quartiles, and median provided.

### 3.2 Multilevel model building

To evaluate the degree of clustering within our data, we first estimated the intraclass correlation coefficient (ICC) from the null random-intercept model, which included no predictors. In this model (Equation 1),


$$\hat{\text{understanding}}=0.632+u_{0j}$$ (1)

the subscript *j* denotes the individual (cluster), and $u_{0j}$ represents the person-specific random intercept. The ICC was 0.211 (95% CI [0.145, 0.296]), indicating that 21.1% of the total variability in understanding scores was attributable to between-individual differences, reflecting meaningful clustering in the data. To test the null hypothesis that there is no variation in understanding scores between individuals, we performed a likelihood ratio (LR) test comparing the null random-intercept logistic model to a single-level logistic regression model. The LR test was significant ( $\chi^{2}$ (1) = 95.25, *p* < 0.001), indicating that the null hypothesis could be rejected and confirming the appropriateness of a multilevel model.

To determine whether adding emotion type as an L1 predictor improved model fit over the null model, we estimated a random-intercepts logistic model with group-mean centered and standardized emotion-type variables. Specifically,


$$\begin{array}{l} \hat{\text{understanding}}=0.651-0.280(\mathrm{et}1_{\mathrm{ij}}-{\bar{\mathrm{et}1}}_{j})-0.377\\ (\mathrm{et}2_{\mathrm{ij}}-{\bar{\mathrm{et}2}}_{j})+0.107(\mathrm{et}3_{\mathrm{ij}}-{\bar{\mathrm{et}3}}_{j})+u_{0j}\; \end{array}$$ (2)

where the predictors—positive emotion type ( et1 ), negative emotion type ( et2 ), and confusion emotion type ( et3 )—represent standardized within-individual deviations from each person’s own mean, and $u_{0j}$ denotes the person-specific random intercept. When predictors were held at their centered means, the estimated odds of understanding were 1.918 (95% CI [1.517, 2.424]). Positive emotion type was associated with lower odds of understanding (OR = 0.756, 95% CI [0.650, 0.879], *p* < 0.001), corresponding to a 24.4% decrease in odds for each one-standard-deviation increase relative to a person’s own mean. Negative emotion type was similarly associated with reduced odds of understanding (OR = 0.686, 95% CI [0.571, 0.825], *p* < 0.001), reflecting a 31.4% decrease per standard deviation. Confusion showed no significant association with understanding (OR = 1.11, *p* = 0.26).

Overall, the LR test indicated that adding the emotion-type predictors improved model fit relative to the null model ( $\chi^{2}$ (3) = 45.26, *p* < 0.001). To quantify this improvement, McFadden’s R-squared was 0.0248, indicating a 2.5% increase in log-likelihood compared with the null model. Although McFadden’s R-squared values are typically small, this result reflects a modest improvement in fit attributable to the emotion-type predictors. Although both positive and negative emotion types significantly predicted understanding, their coefficients were not statistically compared because the study was not powered to detect differences between these effects.

We next tested whether trait Conscientiousness improved model fit beyond the L1 predictors. An LR test comparing the model with Conscientiousness (Equation 2) and without it was not significant ( $\chi^{2}$ (2) = 3.05, *p* = 0.22).

To assess whether the association between emotion type and understanding varied by Conscientiousness, we tested a cross-level interaction. The interaction was not significant ( $\chi^{2}$ (8) = 15.43, *p* = 0.051), when compared with the random-intercepts model (Equation 2), so the more parsimonious model was retained as the final model.

**Figure 1:**
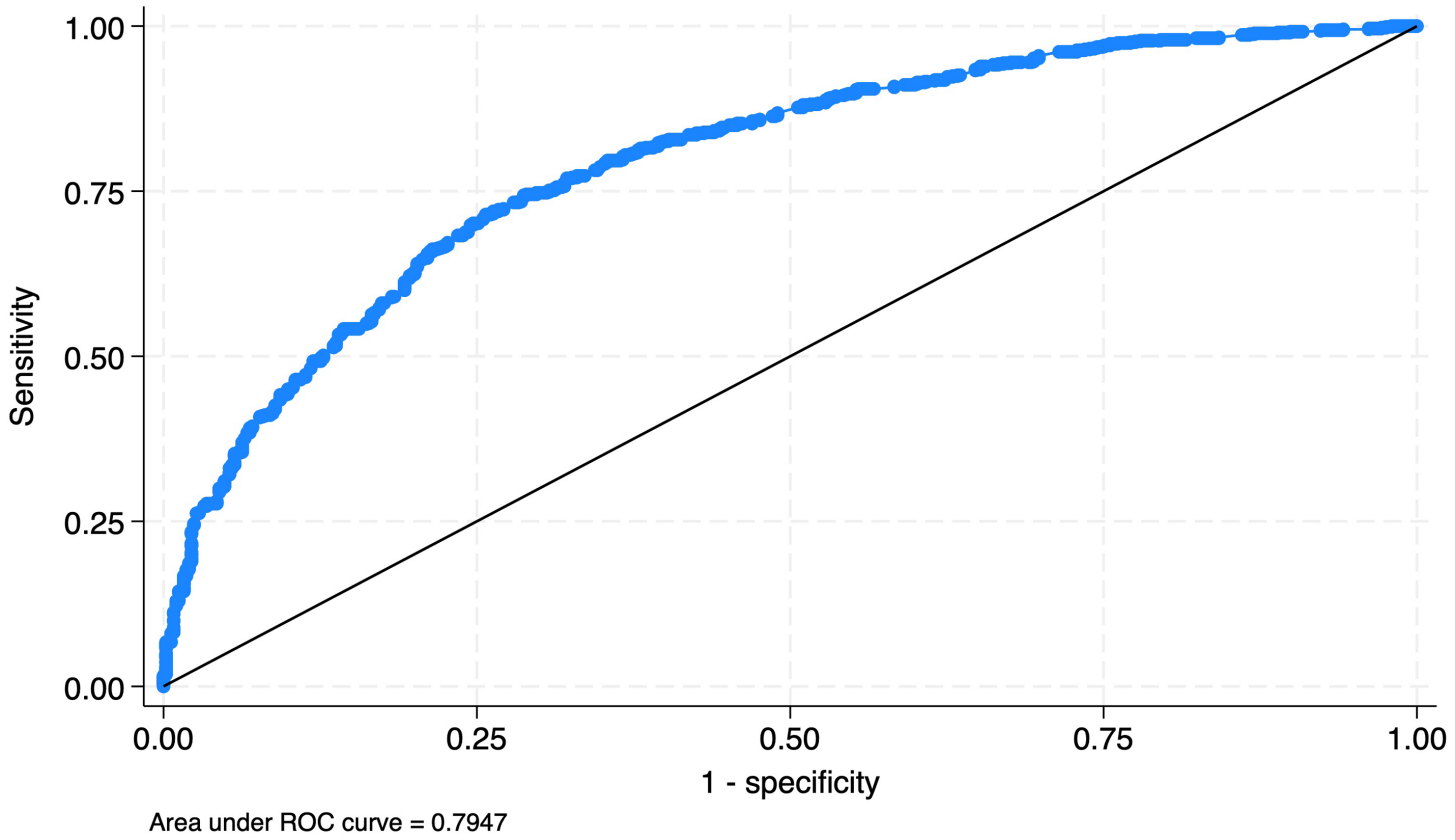
The Receiver Operating Characteristic (ROC) curve of the model. The blue curved line (ROC) represents the performance of our model at all classification thresholds, while the diagonal line represents the performance of a model that classifies outcomes no better than chance. The area under the blue line (AUC = 0.795) indicates that our model has good discriminative ability.

**Figure 2:**
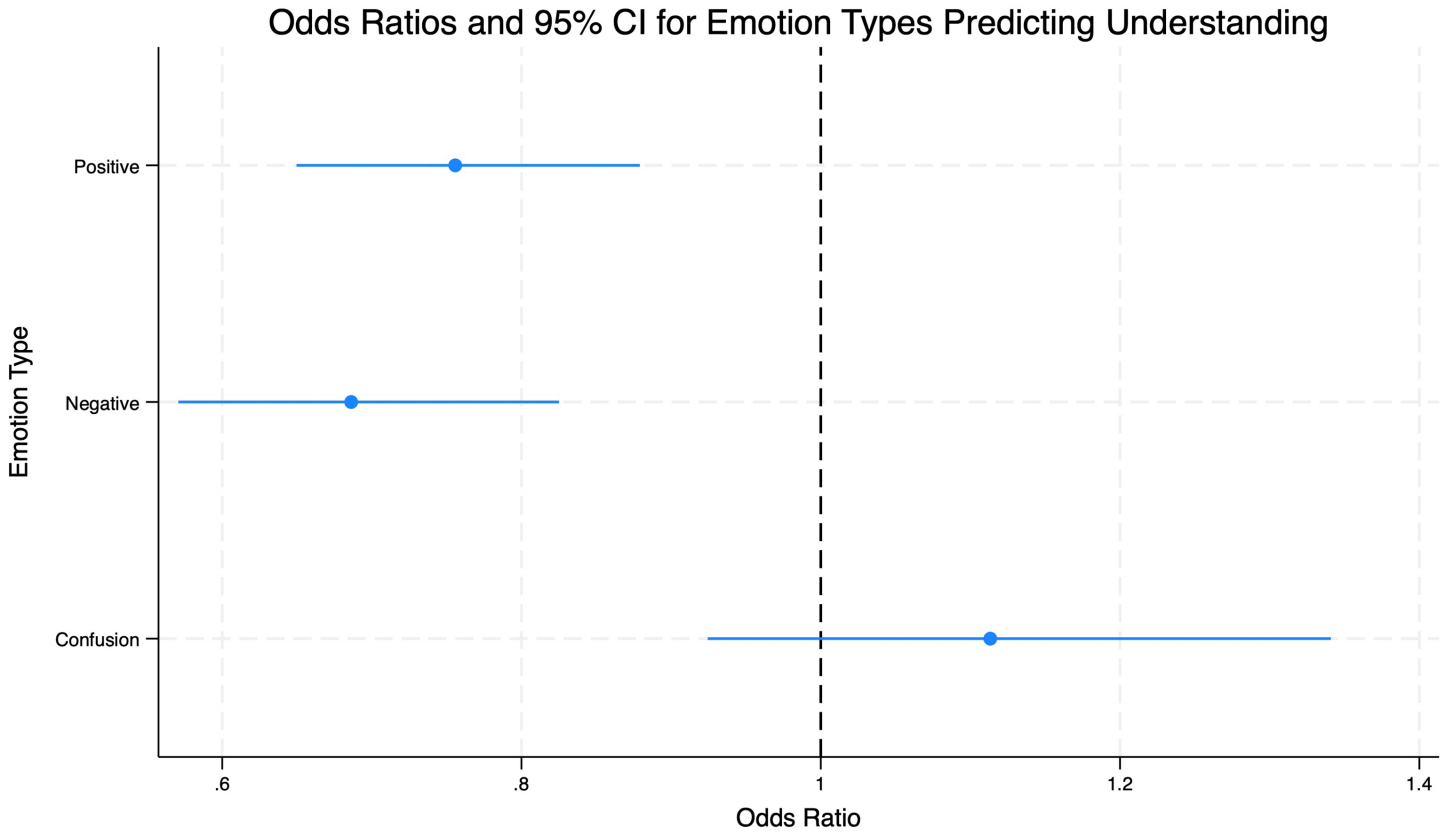
Logistic MLM odds ratios displayed using a dot-and-whisker plot for each emotion type. The *x*-axis represents the effect size in terms of odds ratios, while the *y*-axis represents the three emotion types (independent variables): positive, negative, and confusion. The plot illustrates the significant negative influence of both positive and negative emotions on the likelihood of understanding. Conversely, the effect of confusion does not show a statistically significant impact on understanding.

The area under the ROC curve was 0.795, indicating good discrimination (Figure 1). Effect size estimates and confidence intervals for each emotion type are shown in the dot-and-whisker plot (Figure 2).

## 4 Discussion

The aim of this study was to examine how emotion type is associated with understanding and whether this association is moderated by personality. Using riddle outcome scores, we evaluated the effects of distinct emotion types and trait Conscientiousness on understanding. The finding that 21.1% of the variance in understanding was attributable to between-individual differences supported the use of a logistic multilevel model. At the same time, variability attributable to individuals in repeated-measures designs is often higher, and our ICC may therefore be considered modest for this type of data (Arend and Schäfer, 2019).

While we anticipated directional effects of specific emotion types on understanding, this hypothesis was only partially supported. Negative emotion type (anger, disgust, fear, contempt, and sadness) and positive emotion type (joy) were both significantly associated with lower odds of understanding, whereas confusion showed no association. The negative effect of negative emotion type aligns with prior evidence that negative emotions impede understanding (D’Mello and Graesser, 2012; Gable and Harmon-Jones, 2010; Pekrun et al., 2010). High-intensity negative states, such as disgust, have been shown to constrict attentional focus (Gable and Harmon-Jones, 2010), which can hinder riddle solving by limiting the perceptual flexibility required to shift away from an initially constraining interpretation. Anger, fear, and contempt—similar in intensity and motivational tendencies—may exert comparable effects on understanding.

In contrast, the negative effect of positive emotion type diverges from research showing that positive affect often supports understanding (D’Mello et al., 2017; Pekrun et al., 2011; Schweder, 2019). One possible interpretation is that some participants experienced joy (notable in their obvious facial expressions) at arousal levels high enough to impede performance, consistent with findings that high-arousal positive emotions can reduce effortful control (Valiente et al., 2011). However, the effects of positive and negative emotion type in this study were small. This is reflected in the McFadden’s R-squared value, which indicated that adding emotion-type predictors yielded only a modest improvement (approximately 2.5%) in log-likelihood relative to the null model.

The non-significant effect of confusion may partly reflect its lower frequency and intensity relative to other emotions. As shown in Table 1, confusion occurred less often across participants, reducing variability and signal strength. This uneven distribution likely attenuated statistical power and limited the detectability of its association with understanding, particularly within the brief, low-stakes task context. The non-significant effect of confusion also aligns with previous studies. Given that epistemic emotions have a variable effect on understanding depending on the subsequent cognitive processes stimulated and emotions evoked by these emotions (D’Mello and Graesser, 2012), it is possible that the effect of confusion balanced out in this study. In other words, confusion could have had negative and positive effects on understanding scores depending on their utility per individual, resulting in a non-significant effect overall. The model of affective dynamics proposed by D’Mello and Graesser (2012) illustrates how the experience of confusion can frequently segue into flow and frustration—emotions that, respectively, facilitate and hinder learning in substantial ways.

When the cross-level interactions between emotion type and trait Conscientiousness were examined, no significant results were found. Given the established patterns associated with the Big Five, including Conscientiousness and positive emotions (Livingstone and Srivastava, 2014; Reisenzein et al., 2020), we expected to see moderation between emotion type and trait Conscientiousness, especially since most participants would not have firm beliefs associated with riddle-solving (Pekrun, 2006). However, it is important to note that the lack of significant findings does not negate the importance of personality as a key variable in shaping the association between emotions and understanding. Instead, these results highlight the importance of considering context when planning future studies.

The main findings of this study could be better understood by considering its context. According to the Control-Value Theory (Pekrun, 2006; Pekrun and Perry, 2014), emotions emerge in response to the value a learner assigns to a task and its outcome. It would be reasonable to consider the riddle task as low-stakes and assume that most participants would assign a low extrinsic or intrinsic value to it. Consequently, participants would likely not be emotionally invested, making any emotions evoked relatively low in intensity. This explanation accounts for the overall small significant effect sizes and non-significant findings in our study. If participants were confronted with a high-stakes situation where their understanding scores determined their graduation from university, for example, we would anticipate greater emotional investment and a larger effect. That said, researchers must find creative ways to simulate emotional investment. For example, leveraging financial concerns, such as student debt, could be effective. Offering a high-value incentive, like a $500 gift card with a 2% chance of winning, may increase perceived stakes in a controlled setting.

Importantly, when participants understood a riddle, their understanding could be classified as either ‘established’ or ‘emergent’. In many instances, participants did not struggle with solving the riddles and provided a correct answer and justification almost immediately after reading them. In these cases, their understanding would be considered ‘established’. Conversely, when participants grappled with solving the riddles, taking a relatively longer time or using the entire three-minute window to provide a correct answer and justification, their understanding would be considered ‘emergent’ or experienced as an insight. In cases of emergent understanding, participants appeared to be more overtly emotional compared to cases of established understanding, where participants seemed to have a more subdued or minimal emotional experience.

Established understanding occurred more frequently as participants became familiar with the riddle-solving process. As participants solved more riddles, they reported that their experience with previous riddles enabled them to adopt a perspective or skill that facilitated their ability to solve the riddles, effectively automating the process. This fluency resulted in a reduced need for central processing and less emotional variation. Emotions are integral to effortful learning (D’Mello and Graesser, 2012), and when individuals have high control over a task and assign a low value to it, the emotional experience will be less varied and minimal compared to a situation with high control and high value (Pekrun, 2006). Consequently, the differences in arousal and frequency of emotions evoked between cases of established and emergent understanding are consistent with the study’s context and should be further explored in a follow-up study. This interpretation aligns with recent experimental evidence on externally elicited emotions: Mirabella et al. (2024) and Mirabella and Montalti (2025) demonstrated that emotional stimuli affect behavioural and attentional responses only when they are task-relevant, i.e., when they align with the participant’s goals. This convergence suggests that whether emotions are internally generated, as in the present study, or externally induced, their influence on cognition depends on contextual relevance and goal engagement.

## 5 Limitations

This study faced several limitations. Firstly, our sample size at L1 (1,314 total observations, averaging 13 riddles answered per participant) and L2 (101 clusters) met general recommendations for adequate sampling in a two-level multilevel model (McNeish and Stapleton, 2016). Although our number of clusters provided reasonable statistical power for detecting personality trait effects categorized into tertiles, increasing the number of clusters would enhance the power and generalizability of our findings. A recent study using Monte Carlo simulation (Arend and Schäfer, 2019) suggests that more participants (clusters) should have been recruited at L2, given the small effects observed in our study. With an average of 13 scores per cluster, we had a moderate number of observations within each cluster. Ideally, more observations per cluster would be desired to ensure adequate power for detecting emotion-type associations. Additionally, gender was not controlled for in the multilevel model, and the gender imbalance may have influenced the detection or expression of emotions.

With only one randomized riddle sequence, this study faced potential order effects as a limitation. To minimize this issue, ideally, each participant should receive a unique random order of riddles. Non-unique randomization may lead to learning or fatigue effects, as participants’ performance could be influenced by the position of a riddle within the sequence. For instance, encountering similar or easier riddles early on might affect subsequent performance, while facing more challenging riddles early could result in fatigue or demotivation, impacting performance on later riddles. Employing unique randomization would enable a more comprehensive understanding of how riddle order and difficulty influence participants’ performance. Moreover, because perceived task difficulty was not directly measured or controlled, it may have contributed to variability in emotional responses and understanding scores. Future work should also examine how emotional activation fluctuates across task progression to clarify how emotional states evolve during the understanding process.

Notably, the frequency distribution of each emotion type exhibited a considerable range, marked by a substantial number of outliers present in the data, as depicted in Table 1. The averages of each emotion type were influenced by high-frequency values, distorting the data towards skewness. Furthermore, a median score of zero for every emotion type suggests that at least half of the participants did not experience the measured emotions during their attempts to solve the riddles.

Emotions known to play a significant role in understanding include intrigue, boredom, and frustration. However, these emotions were not detectable options in the analytical software we used (see text footnote 1). Instead, we were limited to the seven emotions the software could detect, which we categorized as positive, negative, or epistemic. The positive and epistemic categories each contained one emotion, while the negative emotion category included five, resulting in a total of seven emotions. A concern with combining multiple negative emotions into a single variable is the potential to obscure the unique effects of each negative emotion on understanding. Different negative emotions might have distinct effects, and by aggregating them, we risk losing valuable information. While we standardized the grouped emotion-type predictors to improve comparability, each negative emotion (anger, sadness, disgust, contempt, and fear) could have been modelled separately. However, doing so would have substantially increased model complexity and required a larger sample size than available. This decision represents a methodological compromise to preserve statistical power and interpretability. A simpler design—using one representative negative, positive, and epistemic emotion—could also have reduced model complexity. Future studies with larger samples could examine the unique effects of individual negative emotions on understanding, if theoretically justified.

Additionally, we did not examine interactions between personality traits, despite individuals possessing multiple personality traits. Such interactions would have added substantial complexity to the model, and our study lacked both the statistical power and theoretical justification to support them. Instead, we focused on the well-established relationship between trait Conscientiousness and academic performance.

## 6 Future directions

Our primary interest lies in understanding how emotions influence the development of insights, ideas, skills, and perspectives, as opposed to automated or easily “attained” understanding. Based on this exploratory study, future research should focus on examining and prioritizing the role of emotions in shaping the process of understanding, with an emphasis on emergent rather than established understanding. The distinction between these two types of understanding is vital, as emergent understanding tends to stimulate emotions and foster emotional engagement more intensely, while established understanding does so to a considerably lesser degree. This highlights a unique dynamic between emotions, understanding, and the interrelated cognitive processes involved in learning. The influence of personality may also differ across these two types of understanding scenarios. Therefore, any theoretical model grounded in this phenomenon must take these variations into account. On a practical level, we recommend the development of specialized teaching strategies and intelligent tutoring systems that draw from our observations and these theoretical underpinnings. For emergent understanding, focusing on students’ emotions and personality may prove effective, while this approach might be less impactful for established understanding. Implementing these customized teaching strategies would necessitate an initial evaluation of students’ foundational knowledge concerning the subject or task to inform the most effective approach for enhancing their learning.

In addition, further research should place emphasis on understanding in authentic, real-world contexts. This can be achieved by incorporating tasks that increase intrinsic or extrinsic value, thereby fostering heightened emotional engagement. The second and third authors are currently conducting a follow-up study on emotions in understanding, using poems instead of riddles. Participants respond to questions of varying difficulty about the poems to assess understanding. This study aims to assess how well the findings generalize across different learning contexts. Furthermore, these studies should explore the potential moderating effects of personality on the interplay between emotions and understanding. It would also be beneficial to consider a broader spectrum of emotions, such as intrigue, boredom, curiosity, contentment, flow, and understanding. In a study designed with these considerations in mind, we anticipate observing a distinct influence of emotions on understanding, with personality acting as a moderating variable.

## 7 Conclusion

Goals orient us towards certain experiences which, in turn, trigger certain emotions. Emotions shape perception and our capacity to understand, aligning with Albert Bandura’s view that ‘Personal goal setting is influenced by self-appraisal of capabilities. The stronger the perceived self-efficacy, the higher the goal challenges people set for themselves and the firmer is their commitment to them’ (Bandura, 1993). This self-understanding (or self-misunderstanding) becomes the foundation of our abilities and external understanding. Hence, emotions serve to not only recalibrate self-appraisals but also significantly impact future performance and overall understanding.

Our study introduces unique theoretical and practical insights drawn from the observation that understanding can be either pre-existing (established) or newly acquired (emergent). It also illuminates two primary insights: (a) the context in which understanding occurs and the emotional engagement that context elicits, and (b) the variability among individuals exhibiting distinct personality traits. We propose the development of theoretical models that distinguish between these two forms of understanding, particularly focusing on their distinct interactions with emotions. While emotional expressions may vary across contexts and individuals, emergent understanding necessitates core processes and emotional drives, such as the tenacity required to navigate through confusion when grappling with a complex problem or concept. In this context, if individual differences, like trait Conscientiousness, can be modelled and separated from the overall emotional expression in a high-stakes understanding task, it could reveal the common threads in emotional experiences leading to emergent understanding. Such insights could pave the way for the creation of technologies specifically engineered to facilitate emergent understanding and foster sustained deep learning.

## Data Availability

The datasets presented in this article are not readily available because the dataset referenced in this article is not openly accessible due to ethical considerations. Participants granted consent for their data, including captured facial expressions, to be exclusively used by the researchers conducting this study, with a commitment to preserving their confidentiality and privacy. Requests to access the datasets should be directed to ML, steven.lazic@mail.utoronto.ca.

## Footnotes

1
https://www.imotions.com

## Appendix A

Riddle 1: I start with M, end with X and have a never-ending amount of letters. What am I? Answer: Mailbox.

Riddle 2: What 5 letter word becomes shorter when you add two letters to it? Answer: Short.

Riddle 3: What building has the most stories? Answer: Library.

Riddle 4: I have two coins that equal 30 cents and one is not a nickel. What two coins do I have? Answer: A quarter and nickel.

Riddle 5: I saw a boat full of people, yet there wasn’t a single person on board. How is this possible? Answer: Everyone on the boat was in a relationship.

Riddle 6: Three different doctors said that Paul is their brother, yet Paul claims he has no brothers. Who is lying? Answer: No one.

Riddle 7: Why is it against the law for a man living in Delhi to be buried in Mumbai? Answer: Because he is alive.

Riddle 8: Before Mt. Everest was discovered, what was the highest mountain in the world? Answer: Mt. Everest.

Riddle 9: A big brown cow is lying down in the middle of a country road. The streetlights are not on, the moon is not out, and the skies are heavily clouded. A truck is driving towards the cow at full speed, its headlights off. Yet the driver sees the cow from afar easily, and avoids hitting it, without even having to brake hard. How is that possible? Answer: It was daytime.

Riddle 10: Which month has 28 days? Answer: Every month has at least 28 days.

Riddle 11: In a lake, there is a patch of lily pads. Every day, the patch doubles in size. If it takes 48 days to cover the entire lake, how long does it take for the patch to cover half of the lake? Answer: 47 days.

Riddle 12: You are a cyclist in a cross-country race. Just before the crossing finish line, you overtake the person in second place. In what place did you finish? Answer: Second.

Riddle 13: A bat and a ball cost $1.10 in total. The bat costs a dollar more than the ball. How much does the ball cost? Answer: 5 cents.

Riddle 14: You’re in a dark room with a candle, a wood stove, and a gas lamp. You only have one match, what do you light first? Answer: The match.

Riddle 15: How much dirt is there in a hole that is 3 feet deep, and 6 inches in diameter? Answer: None.
